# Disorders of the Nervous System in Childhood

**Published:** 1872-01

**Authors:** 


*Disorders of the Nervous System in Childhood.* By Charles West, M. D. Philadelphia, Henry C. Lea, 1871.

This work contains three Lectures. 1st, on Neuralgia and Epilepsy. 2nd, on Chorea and Paralysis. 3d, Disorder and Loss of Power of Speech, Mental and Moral Peculiarities and their disorders. These Lectures were delivered at the Royal College of Physicians of London, in March, 1871. The work is upon subjects in which all physicians are interested, and the lectures will be read with special interest.

